# ED95 determination of continuous norepinephrine infusion for preventing spinal anesthesia-induced hypotension in obese parturients during cesarean delivery

**DOI:** 10.3389/fphar.2026.1905008

**Published:** 2026-08-25

**Authors:** Rui Ran, Limeng Liu, Wenqian Li, Jing Li, Xiaofeng Lei, Jin Yu

**Affiliations:** 1 Department of Anesthesiology, Chongqing Health Center for Women and Children, Women and Children’s Hospital of Chongqing Medical University, Chongqing, China; 2 Department of Anesthesiology, First Clinical College, Chongqing Medical University, Chongqing, China

**Keywords:** cesarean delivery, dose-finding, norepinephrine, obesity, spinal hypotension

## Abstract

**Background:**

Norepinephrine is an effective vasopressor for preventing and managing spinal anesthesia-induced hypotension during cesarean delivery. Nevertheless, data regarding the 95% effective dose (ED_95_) of continuous norepinephrine infusion in obese parturients remain scarce.

**Methods:**

This prospective dose-finding study enrolled parturients scheduled for elective cesarean delivery under spinal anesthesia. Participants were stratified into two groups based on body mass index (BMI): non-obese group (BMI <30 kg/m^2^) and obese group (BMI ≥30 kg/m^2^). Intravenous norepinephrine infusion was initiated at a dose of 0.01 μg/kg/min in the first participant, with subsequent dosages adjusted according to the preceding patient’s response using a biased coin design up-and-down sequential method. Spinal anesthesia-induced hypotension was defined as a mean arterial pressure of below 65 mmHg sustained for more than 1 minute within 30 min following intrathecal injection. The 95% effective dose (ED_95_) was estimated via isotonic regression coupled with bias-corrected bootstrapping. Maternal and neonatal outcomes, including adverse events, were recorded.

**Results:**

Eighty parturients were included in the analysis. The ED_95_ of norepinephrine was determined to be 0.045 μg/kg/min (95% confidence interval [CI], 0.03–0.05 μg/kg/min) in the non-obese group and 0.0662 μg/kg/min (95% CI, 0.055–0.075 μg/kg/min) in the obese group. No significant differences were observed in maternal or neonatal outcomes between the two cohorts.

**Conclusion:**

The findings indicate that obese parturients require a significantly higher prophylactic dose of norepinephrine infusion to prevent spinal anesthesia-induced hypotension compared to the non-obese counterparts. These results underscore the importance of individualized dosing strategies based on BMI to optimize hemodynamic management in obese parturients undergoing cesarean delivery.

## 1 Introduction

Spinal anesthesia-induced hypotension is a frequent complication encountered during cesarean section, particularly among patients with preeclampsia. Severe hypotension may exacerbate uteroplacental hypoperfusion, thereby posing potential risks to fetal wellbeing. The incidence of hypotension associated with spinal anesthesia has been reported to range from 29% to 80%, contingent upon the specific hypotension threshold applied and various risk factors (Burns et al., 2001; Guo et al., 2022; Yu et al., 2021).

Phenylephrine has traditionally been employed both prophylactically and therapeutically to manage spinal anesthesia-induced hypotension; however, its pure α-adrenergic agonist properties can precipitate reflex bradycardia and a consequent reduction in cardiac output (Guo et al., 2022; Zhang et al., 2024; Wang et al., 2020). In contrast, norepinephrine, which exerts α1-mediated vasoconstriction alongside mild β_1_ activity, may confer advantages by preserving maternal heart rate and cardiac output, thereby potentially mitigating the risk of fetal acidosis (Guo et al., 2022; Singh et al., 2026). Multiple randomized controlled trials have substantiated the efficacy and safety of norepinephrine infusions during spinal anesthesia for cesarean delivery (Sharkey et al., 2019; Theodoraki et al., 2020; Wang et al., 2018).

Prophylactic norepinephrine infusion has demonstrated effectiveness in preventing hypotension within the general obstetric population, with investigations exploring both weight-adjusted and fixed-rate dosing regimens (Fu et al., 2020). Accordingly, international consensus guidelines advocate for the use of vasopressors possessing β-agonist activity as the preferred pharmacological intervention for managing hypotension during cesarean section, given their capacity to minimize reflex bradycardia and reductions in cardiac output (Kinsella et al., 2018).

Obese parturients, relative to those with normal body mass index (BMI), exhibit an elevated risk of severe and refractory hypotension, attributable to factors such as aortocaval compression and altered hemodynamic reserve (Mace et al., 2011; Nivatpumin et al., 2023). The physiological differences in cardiovascular responses and pharmacokinetics between obese and non-obese populations necessitate individualized dosing strategies to avoid insufficient prophylaxis or excessive vasopressor exposure. With the increasing prevalence of obesity among women of reproductive age, it is imperative to establish the optimal prophylactic infusion dose of norepinephrine for obese parturients. The presented study seeks to compare the dose requirements of prophylactic norepinephrine infusion for the prevention of spinal anesthesia-induced hypotension during cesarean section between obese and non-obese parturients.

## 2 Materials and methods

This study was approved by the Institutional Review Board of Chongqing Health Center for Women and Children, Chongqing, China on 10 October 2025 (Approval Number: 2025095). Written informed consent was obtained from all participants. The trial was registered prospectively on Chictr.org.cn (Registration Number: ChiCTR-2600117075) prior to patient enrollment and was carried out between January 2026 and April 2026.

The inclusion criteria encompassed parturients aged between 18 and 45 years who were scheduled to undergo elective cesarean section under combined spinal-epidural anesthesia, with an American Society of Anesthesiologists (ASA) physical status classification of II to III. The exclusion criteria were as follows: (1) a documented history of hypersensitivity to vasoactive agents; (2) the presence of preoperative hypertension or eclampsia; (3) preterm parturients with a gestational age of less than 37 weeks; (4) significant preoperative hepatic or renal dysfunction identified prior to surgery; (5) severe thrombocytopenia; (6) ASA physical status of IV or higher; (7) confirmed severe fetal systemic disease before the procedure; and (8) preexisting psychiatric conditions, including delirium or cognitive impairment.

Parturients were enrolled consecutively and stratified into two groups based on their BMI: Group A (non-obese, BMI <30 kg/m^2^) or Group B (obese, BMI ≥30 kg/m^2^). Obesity was defined in accordance with the World Health Organization’s (WHO) criteria for overweight and obesity, specifying a BMI threshold of ≥30 kg/m^2^. The study medication was prepared by a nurse who was not involved in data collection to ensure blinding. Specifically, norepinephrine was prepared by the nurse from a 2 mg stock solution to a total volume of 500 mL, resulting in a final concentration of 4 μg/mL. The prepared solution was utilized within 4 hours of preparation and shielded from ambient light throughout the infusion period. Additionally, the infusion pump was positioned outside the direct line of sight of the data collector to maintain blinding. All participants adhered to standard preoperative fasting protocols.

Prior to surgery, written informed consent was obtained from the patient for both anesthesia and participation in the study protocol. An intravenous catheter was inserted into the left upper limb before the patient entered the operating room. Upon arrival, standard monitoring procedures were initiated, including electrocardiography, peripheral oxygen saturation (SpO_2_), and automated noninvasive blood pressure measurements taken from the right upper arm at 2-min intervals. The patient was positioned in the lateral decubitus posture, and a diluted norepinephrine solution was connected for intravenous infusion administration via a SLGO CP-730TCI dual-channel target-controlled infusion syringe pump (Beijing SLGO Medical Technology Co., Ltd., Beijing, China). Combined spinal-epidural anesthesia was administered by an experienced anesthesiologist. Following identification of the epidural space at the L3-4 interspace using an 18-gauge epidural needle, a 25-gauge spinal needle was carefully advanced through the epidural needle to puncture the dura mater, with confirmation of cerebrospinal fluid reflux. After verifying free cerebrospinal fluid flow, 3 mL of 0.75% hyperbaric ropivacaine (mixed with 1 mL of 5% glucose solution) was injected intrathecally. Simultaneously, norepinephrine infusion was commenced via the syringe pump. The spinal needle was then withdrawn, and an epidural catheter was threaded through the epidural needle. Subsequently, the patient was repositioned supine with a 15-degree left lateral tilt of the operating table. Intermittent manual uterine displacement was also performed prior to the initiation of surgery to mitigate aortocaval compression caused by the gravid uterus. Sensory blockade was assessed using a sterile needle and confirmed to reach a level no lower than T6.

The initial norepinephrine dose (Noradrenaline Bitartrate Injection, Jinyao Heping [Tianjin] Pharmaceutical Co., Ltd., National Drug Approval Number: H12020621, Batch No.: 2510281.) was set at 0.01 μg/kg/min, calculated according to actual body weight (ABW). Dose increments of 0.01 μg/kg/min were determined based on previous literature (Tan et al., 2024) and preliminary pilot studies. Spinal anesthesia-induced hypotension was defined as a mean arterial pressure (MAP) below 65 mmHg sustained for more than 1 minute within 30 min following intrathecal administration. In cases where hypotension occurred, the norepinephrine dose was increased by 0.01 μg/kg/min for the subsequent patient. Conversely, if hypotension was not observed, indicating that the prophylactic norepinephrine dose was adequate, the next patient was randomized to receive either the same norepinephrine dose (with a 95% probability) or a reduced dose by 0.01 μg/kg/min (with a 5% probability), employing the biased coin design up-and-down sequential method.

Blood pressure (BP), heart rate (HR), and SpO_2_ were measured at baseline—defined as after 3 minutes in the supine position within the operating room, and subsequently at 2-min intervals until the completion of the surgical incision closure. Intraoperative maternal hypotensive episodes, characterized by a MAP below 65 mmHg, along with other adverse events, including nausea, vomiting, and sinus bradycardia (HR less than 60 beats per minute), were recorded. Additionally, intraoperative parameters, including blood loss, amniotic fluid volume, volume of fluid resuscitation, and urine output were documented. Neonatal outcomes were also assessed, encompassing sex, birth weight, Apgar scores at 1, 5, and 10 min post-delivery, umbilical cord blood gas analyses, and neonatal admission to the neonatal intensive care unit (NICU). All data collection was performed by an anesthesiologist who remained blinded to the drug administration protocol.

### 2.1 Outcomes

The primary objective of the study was to determine the ED95 determination of norepinephrine infusions for the prevention of spinal anesthesia-induced hypotension, utilizing a dose-response curve analysis. The ED95 value was estimated through isotonic regression, accompanied by bias-corrected 95% confidence intervals (CI) derived via bootstrapping methods (Pace and Stylianou, 2007; Stylianou et al., 2003; Stylianou and Flournoy, 2002). To ensure monotonicity in the response probabilities, the pooled adjacent violators algorithm (PAVA) was applied to obtain the adjusted response probability (Tan et al., 2024; Stylianou et al., 2003; Belin et al., 2023). Additionally, the study evaluated secondary outcomes encompassing maternal, fetal, and hemodynamic parameters. Maternal outcomes included the incidence of bradycardia as well as nausea and/or vomiting, while fetal outcomes were assessed through Apgar scores and the necessity for NICU admission.

### 2.2 Sample size estimation

The optimal sample size for the biased coin design with the up-and-down sequential methodology remains undetermined. In the context of anesthesia research, investigations employing the up-and-down approach commonly include between 20 and 40 participants (Pace and Stylianou, 2007). Drawing upon previous experience with this method (Chen et al., 2021; Xiang et al., 2021), a sample size of 40 patients per group is considered relatively substantial in the dose-finding literature. Consequently, this study enrolled a total of 80 parturients, with 40 individuals allocated to each group.

### 2.3 Statistical analysis

The normality of continuous variables was assessed using the Kolmogorov-Smirnov test. For data exhibiting a normal distribution, between-group comparisons were performed using the independent-samples t-test, with results expressed as mean ± standard deviation. Data not conforming to normality were reported as the median [interquartile range (IQR)] and analyzed via the Mann-Whitney U test. Categorical variables were compared using the chi-square test and are presented as frequencies and percentages (n [%]). Statistical analyses were performed using R software version 4.0 (R Foundation for Statistical Computing, Vienna, Austria) and IBM SPSS Statistics version 23.0 (IBM SPSS, Inc., Chicago, IL). Dose-response curves were generated using R. A significance threshold of P < 0.05 was adopted for all statistical tests.

## 3 Results

**FIGURE 1:**
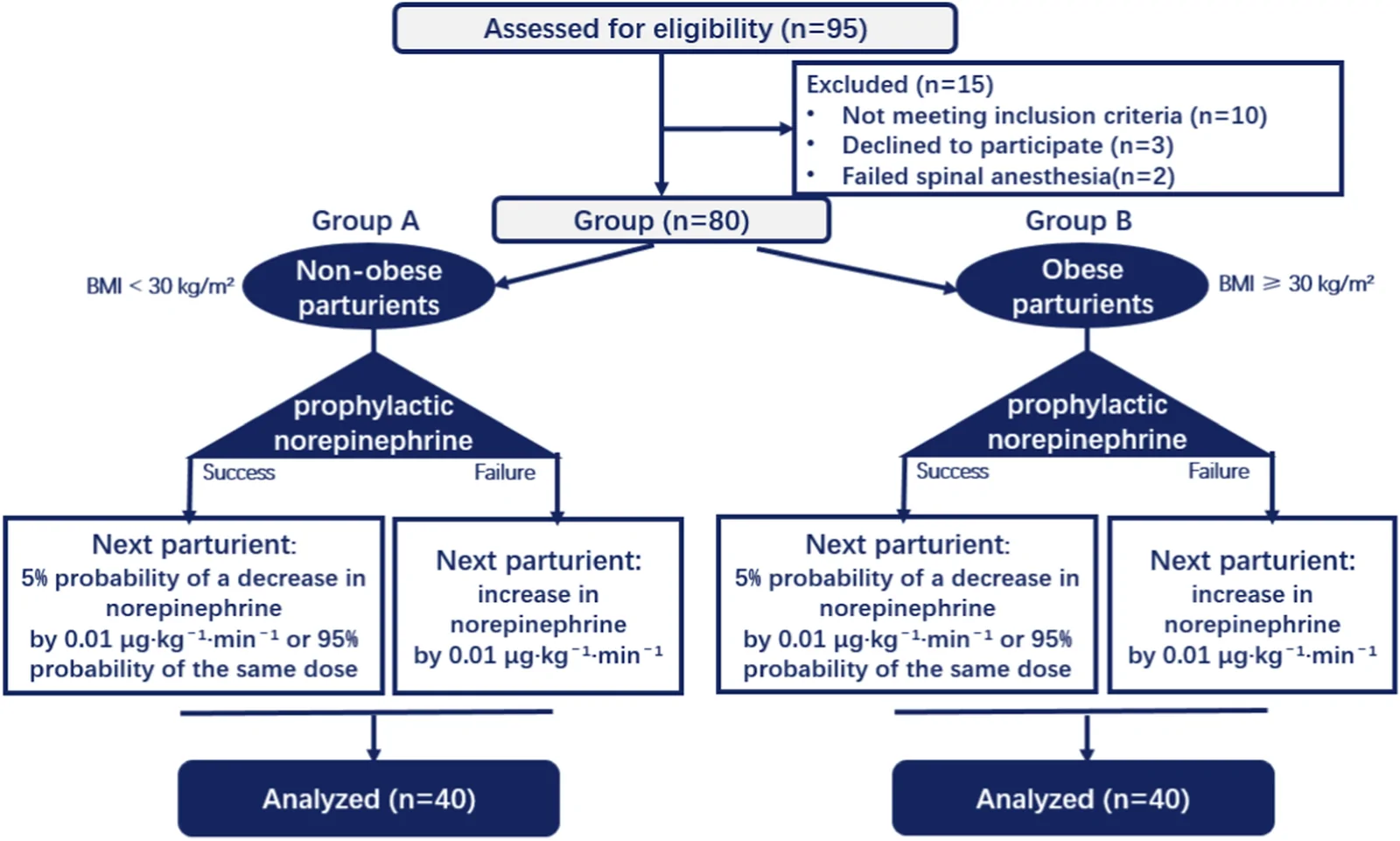
Consolidated standards of reporting trials (CONSORT) flowchart showing patient recruitment.

In this prospective study, a total of 80 parturients were recruited, as illustrated in Figure 1. Maternal characteristics, including age, height, baseline blood pressure, baseline heart rate, sensory block level, and the incidence of cesarean deliveries, did not exhibit statistically significant differences between the two groups, as detailed in Table 1. Consistent with the grouping criteria, body weight, BMI, and abdominal circumference were significantly greater in Group B compared to Group A (*P* < 0.001).

**TABLE 1:** Baseline maternal characteristics and surgical data.

Parameters	Non-obese group (n = 40)	Obese group (n = 40)	*P* value	95% CI
Age, years	31.15 ± 4.57	31.40 ± 4.28	0.801	−2.22–1.72
Height, cm	158.58 ± 4.99	158.17 ± 4.79	0.716	−1.78–2.58
Weight, kg	65.88 ± 5.81	80.61 ± 6.55	<0.001	−17.49∼ −11.97
Body mass index, kg/m^2^	26.30 ± 2.35	32.24 ± 1.61	<0.001	−6.85∼ −5.05
Abdominal circumference, cm	99.55 ± 5.03	110.68 ± 5.26	<0.001	−13.42∼ −8.83
Cesarean section frequency	0 (0–2)	0 (0–2)	0.850	−0.29–0.24
Baseline systolic blood pressure, mmHg	126.60 ± 14.96	130.82 ± 9.91	0.140	−9.87–1.42
Baseline diastolic blood pressure, mmHg	80.68 ± 11.46	82.90 ± 10.43	0.367	−7.10–2.65
Baseline mean arterial pressure, mmHg	100.83 ± 14.67	102.55 ± 10.57	0.548	−7.42–3.97
Baseline heart rate, bpm	93.40 ± 14.73	98.90 ± 12.32	0.074	−11.55–0.55
Sensory block	T6 (T4-T6)	T6 (T4-T6)	0.370	—

Data are presented as mean ± SD or median (IQR); Abbreviations: SD, standard deviation.

All participants included in the analysis underwent a combined spinal-epidural anesthesia procedure, with an epidural catheter inserted as a contingency measure. Notably, no supplementary local anesthetics were administered via the epidural catheter during the cesarean section procedure.

**FIGURE 2:**
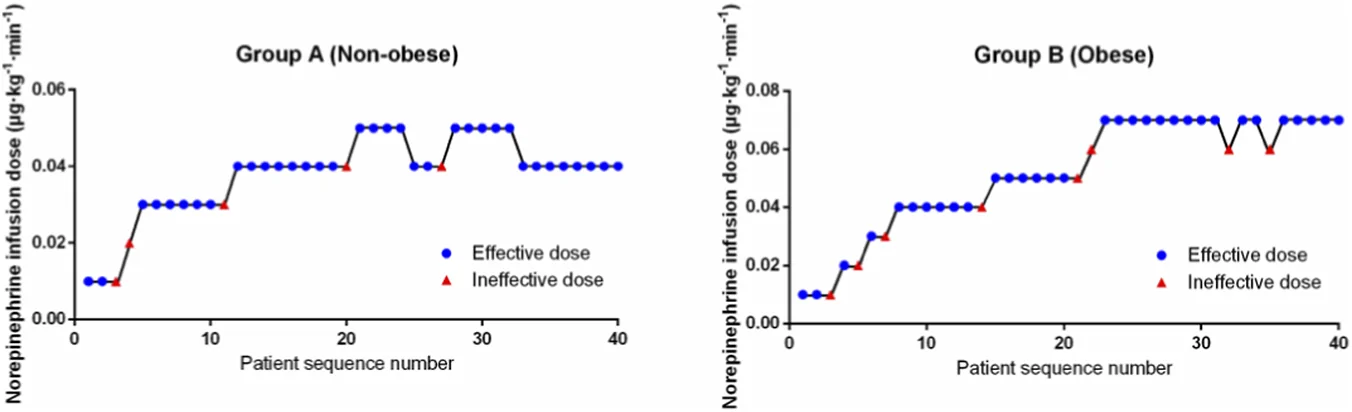
Process for determining the 95% effective dose (ED_95_) of norepinephrine. The graph displays the parturient sequence number on the x-axis and the corresponding norepinephrine infusion dose on the y-axis. Effective doses are represented by filled circles (●), whereas ineffective doses are denoted by filled triangles (▲).

**FIGURE 3:**
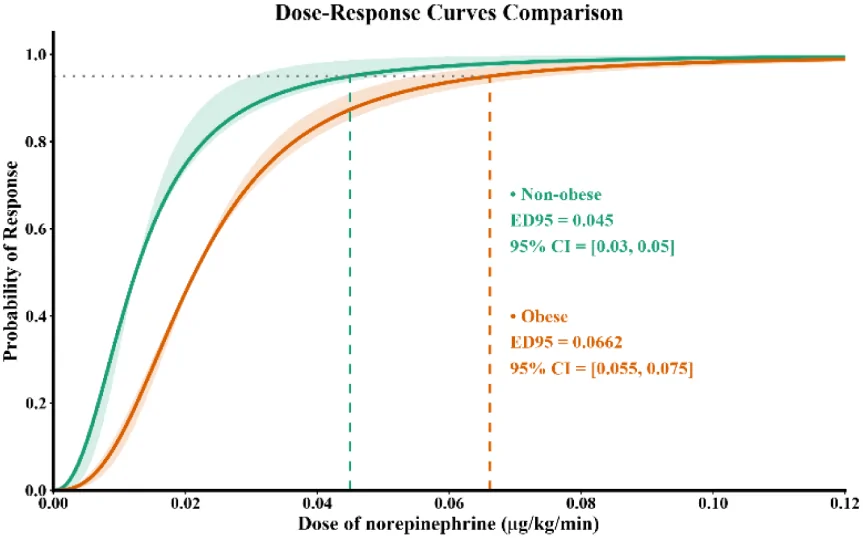
Dose-response curve of continuous norepinephrine infusion for preventing spinal anesthesia-induced hypotension in obese and non-obese parturients undergoing cesarean delivery. The ED_50_ and ED_95_ for non-obese parturients were determined to be 0.0125 μg/kg/min (95% CI, 0.01–0.04 μg/kg/min) and 0.045 μg/kg/min (95% CI, 0.03–0.05 μg/kg/min), respectively. In contrast, for obese parturients, the ED_50_ and ED_95_ were 0.0215 μg/kg/min (95% CI, 0.015–0.055 μg/kg/min) and 0.0662 μg/kg/min (95% CI, 0.055–0.075 μg/kg/min), respectively.

The sequential responses to norepinephrine infusion in two groups were displayed in Figure 2: those experiencing spinal anesthesia-induced hypotension (red triangles) and those without it (blue circles). The frequency of norepinephrine dose assignments along with the observed and PAVA-adjusted response rates for participants in the non-obese and obese groups are presented in Table 2 and 3 respectively. The dose-response curves for both groups are depicted in Figure 3. Using isotonic regression analysis, the estimated ED_95_ of norepinephrine was 0.045 μg/kg/min (95% confidence interval [CI], 0.03–0.05 μg/kg/min) for non-obese parturients and 0.0662 μg/kg/min (95% CI, 0.055–0.075 μg/kg/min) for obese parturients. The estimated ED_50_ of norepinephrine was 0.0125 μg/kg/min (95% CI, 0.01–0.04 μg/kg/min) for non-obese parturients and 0.0215 μg/kg/min (95% CI, 0.015–0.055 μg/kg/min) for obese parturients.

**TABLE 2:** Probability and PAVA-adjusted for effective response with norepinephrine in the non-obese group (isotonic regression method).

Dose of norepinephrine (μg/kg/min)	Probability of effective response	PAVA
0.01	0.67	0.50
0.02	0.00	0.65
0.03	0.86	0.86
0.04	0.90	0.90
0.05	1.00	1.00

Abbreviations: PAVA, pool adjacent violators algorithm.

**TABLE 3:** Probability and PAVA-adjusted estimates of effective response to norepinephrine in the obese group (isotonic regression method).

Dose of norepinephrine (μg/kg/min)	Probability of effective response	PAVA
0.01	0.67	0.57
0.02	0.50	0.58
0.03	0.50	0.62
0.04	0.86	0.67
0.05	0.86	0.74
0.06	0.00	0.87
0.07	1.00	1.00

Abbreviations: PAVA, pool adjacent violators algorithm.

There were no significant differences between the two groups in maternal outcomes, including spinal anesthesia-induced hypotension, nausea or vomiting, and bradycardia, as well as in neonatal outcomes, including umbilical artery pH, PaO_2_, PaCO_2_, base excess values (BE) values, and Apgar scores at 1, 5, and 10 min, as detailed in Table 4. Intraoperative parameters, including blood loss, amniotic fluid volume, urine output, and intravenous fluid volume, were also comparable between the two groups (P > 0.05). All newborns in both groups were delivered in stable clinical condition, with none requiring admission to the NICU. Additionally, no neonate required respiratory support, and there were no occurrences of neonatal mortality.

**TABLE 4:** Maternal and neonatal outcomes.

Outcome	Non-obese group (n = 40)	Obese group (n = 40)	*P* value	95% CI
Intravenous fluid volume, ml	355.00 ± 81.49	372.00 ± 117.57	0.455	−62.03–28.03
Amniotic fluid volume, ml	498.75 ± 214.98	540.00 ± 311.98	0.493	−160.51–78.01
Urine volume, ml	135.50 ± 61.14	130.00 ± 74.56	0.719	−24.86–35.85
Blood loss, ml	347.50 ± 59.86	375.00 ± 80.86	0.088	−59.07–4.17
Anesthesia-induced hypotension, n (%)	4 (10.00)	7 (17.50)	0.330	—
Nausea or vomiting, n (%)	7 (17.50)	10 (25.00)	0.412	—
Bradycardia, n (%)	3 (7.50)	1 (2.50)	0.305	—
Infant weight, kg	3.23 ± 0.34	3.46 ± 0.36	0.004	−0.38∼ −0.07
pH	7.28 ± 0.05	7.28 ± 0.05	0.967	−0.02–0.02
PaO_2_, mmHg	38.78 ± 20.91	35.03 ± 11.82	0.327	−3.81–11.31
PaCO_2_, mmHg	49.93 ± 8.52	49.80 ± 8.09	0.947	−3.57–3.82
BE, mmol/L Apgar score (1 min) Apgar score (5 min)	−4.72 ± 2.01 10 (9–10) 10	−4.12 ± 1.64 10 (8–10) 10	0.147 0.656 1	−1.41–0.22 −0.09–1.36 —
Apgar score (10 min)	10	10	1	—

Data are presented as mean ± SD, number (percentage), or median with interquartile range.

## 4 Discussion

The principal finding of this study indicates that obese parturients necessitate a significantly higher prophylactic infusion dose of norepinephrine (ED_95_: 0.0662 μg/kg/min) to effectively prevent spinal anesthesia-induced hypotension, in comparison to their non-obese counterparts (ED_95_: 0.045 μg/kg/min). This disparity can be attributed to several physiological mechanisms. Firstly, the increased abdominal circumference characteristic of obese parturients results in more substantial mechanical compression of the aorta and inferior vena cava, thereby causing a more pronounced reduction in venous return following spinal anesthesia (Nivatpumin et al., 2023). Secondly, obesity-related alterations in body composition, notably augmented adipose tissue, modify the volume of distribution and plasma protein binding dynamics (Smit et al., 2018), which directly impact the dosing requirements of vasoactive agents. Thirdly, obesity is associated with an elevated baseline sympathetic tone (da Silva et al., 2009; Kalil and Haynes, 2012) and potential vasopressor resistance, necessitating a greater degree of vasoconstriction to sustain adequate arterial pressure. It’s important to note that the isolated ED95 value alone is unlikely to dictate distinct clinical decisions; rather, the practical advantage may lie in the cumulative dose-sparing effect observed throughout the intraoperative period. Consequently, the difference in ED95 should be regarded as a pharmacodynamic reference point rather than a directive for implementing substantially different fixed-dose regimens.

Spinal anesthesia administered for cesarean section frequently results in hypotension, which may induce maternal discomfort and fetal hypoxia. Timely intervention is critical to avert severe complications (Lim et al., 2018). Prophylactic management of hypotension has been demonstrated to be more efficacious than reactive approaches. The initiation of a low-dose vasopressor infusion prior to the onset of vasodilation can effectively mitigate the occurrence of hypotension. Additionally, obese parturients exhibit distinct physiological responses and possess an elevated risk of developing hypotension following spinal anesthesia (Catalano and Shankar, 2017). Consequently, individualized dosing protocols for this population are imperative to achieve optimal clinical outcomes.

Numerous investigations have evaluated varying dosages of norepinephrine and phenylephrine for the prevention of spinal anesthesia-induced hypotension in parturients undergoing cesarean section. One randomized controlled trial demonstrated that prophylactic infusions of phenylephrine at 0.5 μg/kg/min and norepinephrine at 0.08 μg/kg/min produced comparable effects on fetal heart rate and maternal cardiac output following spinal anesthesia, without eliciting clinically significant adverse impacts on fetal circulation or neonatal outcomes (Sun et al., 2024). Another study assessed norepinephrine initiated at 0.05 μg/kg/min versus phenylephrine at 0.5 μg/kg/min, with both agents manually titrated during spinal anesthesia for cesarean delivery. Findings indicated that norepinephrine was associated with a higher cardiac index, while both vasopressors effectively maintained maternal blood pressure (Belin et al., 2023). Regarding norepinephrine dosing, Hasanin AM et al. reported that infusion rates of 0.050 μg/kg/min and 0.075 μg/kg/min were more efficacious in reducing the incidence of spinal anesthesia-induced hypotension during cesarean delivery compared to a lower rate of 0.025 μg/kg/min (Hasanin et al., 2019).

For parturients with preeclampsia, the prevention of spinal anesthesia-induced hypotension presents a significant clinical challenge. A comparative study evaluating a higher infusion dose of phenylephrine (0.625 μg/kg/min) against the standard norepinephrine infusion rate (0.05 μg/kg/min) demonstrated comparable incidences of maternal hypotension and neonatal outcomes among preeclamptic patients under spinal anesthesia. However, the administration of phenylephrine was correlated with an increased incidence of bradycardia (Guo et al., 2022). Furthermore, the effective dose for 90% prevention (ED90) of spinal anesthesia-induced hypotension using prophylactic norepinephrine infusion in this patient population was established at 0.054 μg/kg/min (95% CI: 0.053–0.056 μg/kg/min) through isotonic regression analysis (Tan et al., 2024).

The optimal norepinephrine dosage for obese parturients remains unclear. This study sought to establish the effective dose of norepinephrine required to prevent spinal anesthesia-induced hypotension in 95% obese parturients, with a comparative analysis involving non-obese parturients. Findings revealed that obese parturients necessitate higher norepinephrine doses, thereby providing valuable insights for individualized dosing regimens.

Concerns have been raised regarding the potential adverse neonatal effects associated with norepinephrine administration. A randomized controlled trial demonstrated that prophylactic infusions of norepinephrine at 0.08 μg/kg/min and phenylephrine at 0.5 μg/kg/min, administered at equipotent doses, exerted comparable effects on fetal cerebral perfusion (Liu et al., 2025). Furthermore, among parturients undergoing elective or non-elective cesarean delivery under spinal or combined spinal-epidural anesthesia, norepinephrine was found to be non-inferior to phenylephrine with respect to neonatal outcomes, as measured by umbilical arterial pH (Ngan et al., 2020). Another prophylactic regimen indicated that norepinephrine infusion at 6 μg/min was non-inferior to phenylephrine infusion at 80 μg/min concerning umbilical arterial base excess values, with similar incidences of fetal acidosis between the two protocols (Goel et al., 2026). The most recent systematic review and network meta-analysis further corroborated the safety profile of norepinephrine regarding fetal and maternal outcomes in cesarean deliveries (Singh et al., 2026). Collectively, these data provide robust evidence supporting the safety of norepinephrine for the prevention of spinal anesthesia-induced hypotension. The presented study additionally confirmed the efficacy of norepinephrine in preventing spinal anesthesia-induced hypotension for cesarean section in both obese and non-obese parturients, with comparable maternal and neonatal outcomes observed across groups.

Considering the pharmacological characteristics of vasoactive agents, continuous infusion is often regarded as the preferred method of administration. A network meta-analysis encompassing 74 randomized controlled trials with a total of 7,798 patients demonstrated that prophylactic continuous infusion of norepinephrine or phenylephrine is more effective than bolus administration in preventing spinal anesthesia-induced hypotension and in reducing intraoperative nausea and vomiting during cesarean section (I et al., 2025). Furthermore, in high-risk patients undergoing non-cardiac surgery with continuous arterial blood pressure monitoring, continuous norepinephrine infusion during the induction of general anesthesia was shown to enhance blood pressure stability compared to repeated manual bolus dosing (Vokuhl et al., 2025). Although direct comparative studies between intermittent bolus and continuous infusion for managing hypotension following spinal anesthesia during cesarean section are lacking, current guidelines explicitly recommend continuous infusion of norepinephrine over repeated bolus administration, particularly in high-risk patient populations (Pastene et al., 2026).

In this study, obesity was defined according to the WHO criterion, characterized by a BMI exceeding 30 kg/m^2^. It is acknowledged that physiological fluid retention is common in this population and may result in either overestimation or underestimation of true adiposity. Nevertheless, given that the Chinese obesity threshold is generally set at a BMI of 28 kg/m^2^ or higher, the adoption of the more stringent WHO cutoff (≥30 kg/m^2^) constitutes a conservative and methodologically rigorous approach for the present analysis. While ABW is both practical and pharmacokinetically appropriate for norepinephrine dosing, clinicians should exercise heightened caution when selecting initial doses in cases of extreme obesity (e.g., BMI ≥40 kg/m^2^), due to inter-individual variability in fat distribution, and should implement vigilant hemodynamic monitoring.

This study has several limitations that warrant consideration. First, the exclusion of patients with comorbidities such as gestational hypertension or cardiac disease restricts the generalizability of the findings to a wider population of obese parturients. Future dose-finding investigations should specifically target these subpopulations, including individuals with preeclampsia, cardiac comorbidities, or emergent clinical indications, to ascertain their respective norepinephrine ED95 values and to determine whether alternative weight metrics or dosing protocols are necessary. Second, the use of the biased coin design up-and-down sequential method for estimating the effective dose is constrained by a relatively small sample size and relies on predetermined dose intervals and bias parameters. These factors may affect the precision and stability of the dose estimation, as well as its broader applicability. Third, ethical considerations and the intent to minimize invasive procedures led to the employment of intermittent non-invasive blood pressure monitoring, which may have failed to detect transient hypotensive episodes. Future research incorporating advanced hemodynamic monitoring techniques is essential to refine dosing strategies and to assess fetal outcomes within this specific cohort.

In conclusion, obese parturients require higher doses of norepinephrine to prevent spinal anesthesia-induced hypotension compared to their non-obese counterparts. Tailored dosing regimens are imperative to optimize clinical outcomes in this population.

## Data Availability

The original contributions presented in the study are included in the article/supplementary material, further inquiries can be directed to the corresponding author.
